# Segmental PASI Evaluation Reveals Reduced PUVA Responsiveness of Lower-Limb Psoriasis in Patients with Internal Organ Malignancy

**DOI:** 10.3390/jcm15124525

**Published:** 2026-06-11

**Authors:** Miguel Alpalhão, Joana Antunes, João Augusto Ferreira, René Santus, Paulo Filipe

**Affiliations:** 1 Faculty of Medicine, University of Lisbon, 1649-004 Lisbon, Portugal; 2 Dermatology Research Unit, Gulbenkian Institute of Molecular Medicine, University of Lisbon, 1649-004 Lisbon, Portugal; 3 Clínica Cintramédica Portela de Sintra, 2710-437 Sintra, Portugal; 4 Dermatology Department, Unidade Local de Saúde Santa Maria, 1649-035 Lisbon, Portugal; 5 Département RDDM, Muséum National d’Histoire Naturelle, 75005 Paris, France

**Keywords:** PUVA therapy, psoriasis, treatment outcome, body regions, disease burden

## Abstract

**Background/Objectives:** The management of moderate-to-severe psoriasis in patients with a recent history of internal malignancy is a clinical challenge, as systemic immunosuppressive therapies are often avoided because of concerns about cancer recurrence. While Psoralen and Ultraviolet A (PUVA) photochemotherapy remains a valuable non-immunosuppressive alternative, regional variations in therapeutic response are not well-characterized in this population. This study aimed to evaluate total and segmental Psoriasis Area and Severity Index (PASI) responses to PUVA in patients with chronic plaque psoriasis and recent internal organ malignancy. **Methods:** This prospective, single-center, real-world cohort study enrolled 20 adults with moderate-to-severe chronic plaque psoriasis and a recent (<5 years) diagnosis of internal organ malignancy in complete remission. Participants received oral PUVA three times weekly for up to 30 sessions. Primary and secondary outcomes included changes in total PASI, segmental PASI (head/neck, trunk, upper limbs, and lower limbs), and Dermatology Life Quality Index (DLQI) at baseline, completion of therapy, and 6 months post-treatment. **Results:** PUVA led to a significant reduction in mean total PASI from 18.6 ± 3.2 at baseline to 5.7 ± 6.0 at treatment completion (69% reduction; *p* < 0.001). However, regional responses differed significantly: the head and neck improved the most (80.4%), followed by the trunk (72.2%) and upper limbs (72.3%), while the lower limbs showed the weakest response (59.5%; *p* < 0.001). At baseline, trunk contributed the most to total PASI (38%), while post-treatment, lower-limb lesions accounted for approximately 47% of the remaining total disease burden, showing the highest contribution to total PASI of all body regions. At 6 months, the lower limbs remained the most affected area, with significantly lower improvement (52.9%) compared to other regions. Mean DLQI also improved significantly from 17.2 ± 2.8 to 5.6 ± 2.6 (*p* < 0.001). **Conclusions:** PUVA is an effective and safe treatment for patients with psoriasis and a recent history of malignancy. Nevertheless, lower-limb psoriasis is relatively recalcitrant and contributes disproportionately to residual disease burden and relapse. These findings support the use of regional PASI assessment to guide individualized management and clinical expectations in this complex patient group.

## 1. Introduction

The management of moderate-to-severe psoriasis in patients with a recent history of internal malignancy presents a complex clinical challenge, as systemic immunosuppressive therapies are frequently avoided or deferred due to concerns of cancer recurrence. In this population, therapeutic options are limited and must be utilized with caution to avoid compromising oncological remission. Recent European guidelines emphasize risk-adapted therapeutic decision-making in this setting, recommending cautious or deferred use of systemic immunomodulators according to cancer type, stage, and time since remission [1,2]. Psoralen and Ultraviolet A (PUVA) photochemotherapy remains a valuable non-immunosuppressive alternative, offering well-established efficacy for patients requiring intensive treatment, with manageable long-term safety profile under strict cumulative dose monitoring [3].

Despite its utility, the therapeutic response to PUVA is not uniform across all anatomical regions, and standard global Psoriasis Area and Severity Index (PASI) scoring fails to capture significant regional variations in disease burden. Indeed, clinical evidence suggests that lower-limb psoriasis tends to be more recalcitrant to treatment, with recent biologic-era data identifying the legs as a new difficult-to-treat area and the most persistent site of residual disease [4,5]. Earlier phototherapy work also demonstrated that lesional psoriatic skin, particularly when hyperkeratotic, shows reduced UVA sensitivity and requires higher doses to achieve comparable erythemal and therapeutic responses [6]. These observations support the hypothesis that regional factors modulate treatment responsiveness.

This study sought to characterize the total and segmental PASI responses to PUVA in a prospective cohort of patients with chronic plaque psoriasis and recent internal organ malignancy. By analyzing regional outcomes, we aimed to identify patterns of treatment resistance, particularly in the lower limbs, to better inform individualized management and clinical expectations for this complex patient group.

## 2. Patients and Methods

### 2.1. Study Design and Setting

This prospective, single-center, real-world cohort study was conducted at the Dermatology Department of Unidade Local de Saúde Santa Maria, Lisbon, Portugal, between January 2018 and December 2024. Consecutive eligible patients attending the phototherapy unit were invited to join. The study was approved by the institutional ethics committee (P151/18), and written informed consent was obtained from all participants.

### 2.2. Participants

**Figure 1:**
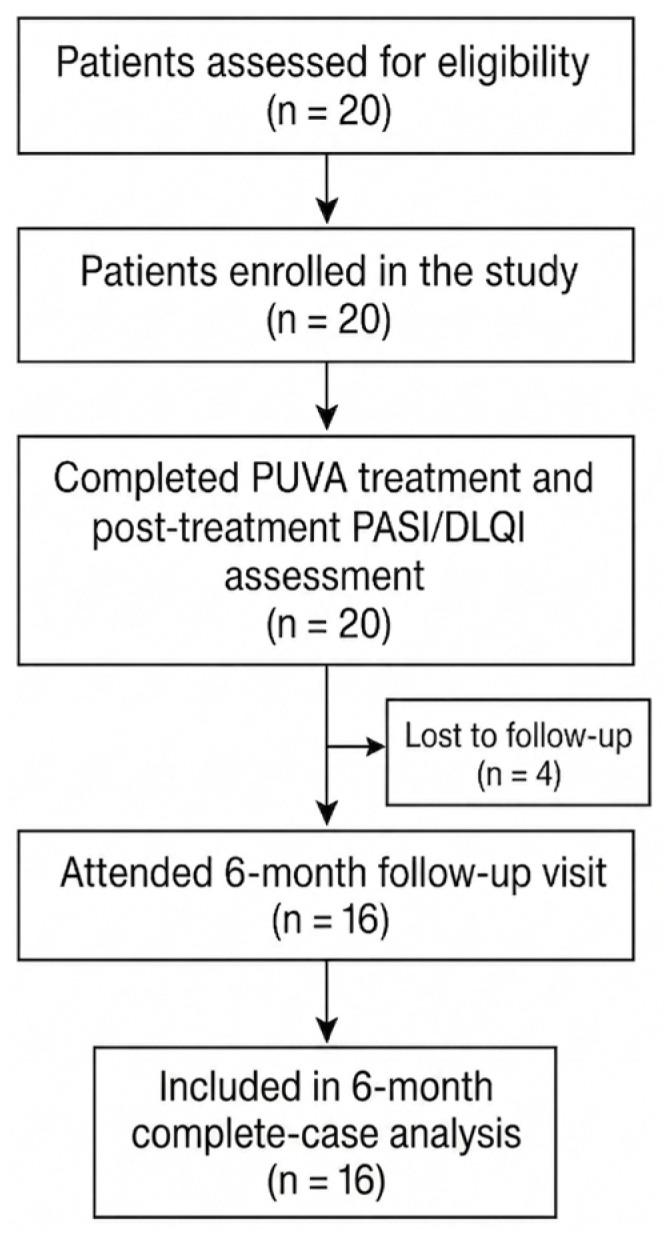
Participant flow diagram. All 20 patients assessed for eligibility were enrolled and completed PUVA treatment with post-treatment PASI and DLQI assessment. Sixteen patients attended the 6-month follow-up visit; four were lost to follow-up.

Adults (≥18 years) with chronic plaque psoriasis (phototype III) and moderate-to-severe disease were eligible if they had a recent (<5 years) diagnosis of internal organ malignancy in complete remission. Exclusion criteria were concomitant systemic antipsoriatic therapy, phototherapy within the previous year, and photosensitivity disorders. Patients were followed from treatment initiation to completion of PUVA and reassessed after 6 months. Reasons for discontinuation and loss to follow-up were recorded (Figure 1).

### 2.3. Intervention

Patients ingested 8-methoxypsoralen (0.6 mg/kg) 2 h before the PUVA session. Using a Daavlin 3 Series NeoLux Plus cabin (Daavlin, OH, USA), Ultraviolet A (UVA) radiation (320–400 nm) was delivered starting at 3 J/cm^2^ with 10–20% increments per session up to 10 J/cm^2^. Treatment was administered three times each week for up to 30 sessions or until clinical plateau. Cumulative UVA dose was recorded. Topical corticosteroids, vitamin D analogs and calcineurin inhibitors, emollients, and keratolytic preparations were allowed during treatment according to the physician’s judgment.

### 2.4. Outcomes and Measurements

The primary outcome was the change in total PASI from baseline to completion of PUVA. Secondary outcomes included PASI75 and PASI90 responses (reductions of 75% and 90% from baseline PASI, respectively), segmental PASI, Dermatology Life Quality Index (DLQI), and response at 6 months. PASI scoring was performed by trained dermatologists using standardized procedures to minimize inter-observer bias. UVA data were obtained from the cabin’s digital log.

### 2.5. Bias and Study Size

Selection bias was reduced by enrolling consecutive eligible patients. Measurement bias was minimized through standardized PASI scoring by the same dermatology team. Due to the exploratory nature of the study, no formal sample size calculation was done. Also, no multivariable adjustment was conducted due to sample size limitations. The sample size reflects the number of eligible patients during the study period.

### 2.6. Statistical Analysis

Continuous variables are presented as mean ± standard deviation (SD) and categorical variables as counts and percentages. Within-patient comparisons were performed using paired Student’s *t*-tests for normally distributed data or Wilcoxon signed-rank tests for non-normal distributions. Between-group comparisons (e.g., moderate vs. severe baseline PASI) were explored descriptively and with independent-samples tests when appropriate. Missing data were handled using complete-case analysis. Statistical significance was defined as two-sided *p* < 0.05. Analyses were performed using IBM SPSS Statistics, version 27.

### 2.7. Ethics

The study was approved by the institutional ethics committee (P151/18) and conducted in accordance with the Declaration of Helsinki. Written informed consent was obtained from all participants.

### 2.8. Use of Artificial Intelligence

**Figure 2:**
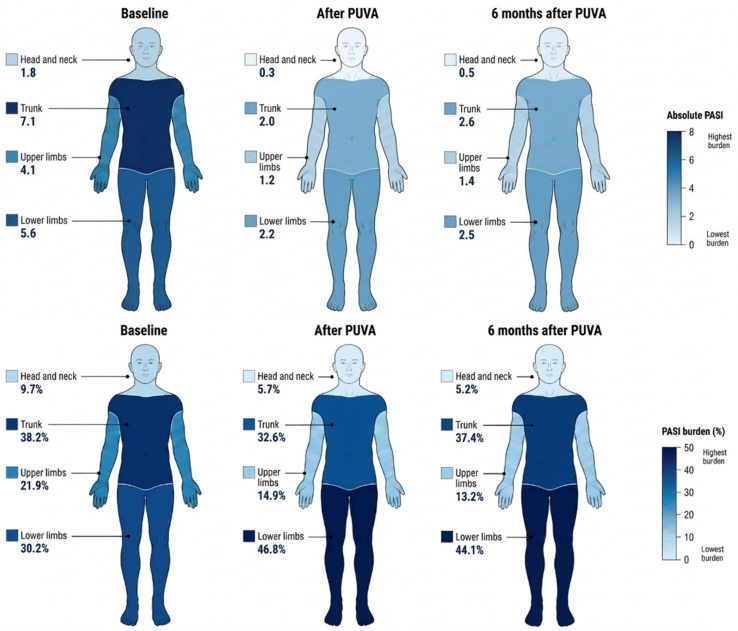
Absolute PASI by body area and time point (**upper panel**) and percentage of total PASI attributable to each body region by time point (**lower panel**). The presented values are the average for each variable. Darker hues represent higher burdens of disease. Despite not having the highest relative contribution to total disease severity, nor the highest absolute segmental PASI at baseline, lower limbs represented the highest absolute PASI among body areas and had the highest relative contribution to total disease activity immediately after PUVA treatment and remained disproportionately represented 6 months after PUVA treatment.

**Figure 3:**
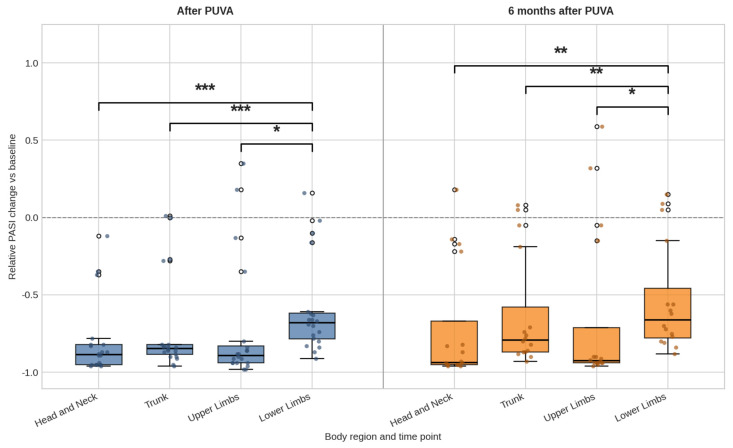
Relative PASI change by body region after PUVA and 6 months after PUVA. Box plots show the distribution of relative PASI change from baseline for the following body regions: head and neck, trunk, upper limbs, and lower limbs, assessed immediately after PUVA and 6 months after PUVA. Each dot represents one patient. The center line indicates the median, the box indicates the interquartile range, and the whiskers represent 1.5× the interquartile range. Brackets indicate pairwise comparisons between each region and the lower limbs within the same time point. Hollow points represent distribution of outliers. Full dots represent each patient. Statistical significance was assessed using two-sided paired Wilcoxon signed-rank tests with Holm correction for multiple comparisons. Significance levels are indicated as follows: *p* < 0.05 (*), *p* < 0.01 (**), and *p* < 0.001 (***). Data were available for 20 patients after PUVA and for 16 patients at 6 months after PUVA.

During the preparation of this manuscript/study the author(s) used Gemini 3.1 for the purposes of improving text fluidity and conciseness. The authors have reviewed and edited the output and take full responsibility for the content of this publication. During the preparation of this manuscript/study the author(s) used GenSpark AI 4.0 for the purposes of improving Figure 2 and Figure 3 graphical quality. The authors have reviewed and edited the output and are fully responsible for the content of this publication. The authors state that the analysis, interpretation, presentation and discussion of the data, other than the reported minor improvements for purposes of clarity, were entirely conducted by the authors.

## 3. Results

### 3.1. Patient Characteristics

Patient characteristics are summarized in Table 1.

Twenty patients were included (mean age 57 years; 55% male). Average psoriasis duration was 14 years (range 4–30). These patients constituted a chronic plaque psoriasis population with long-standing disease and high burden.

All patients in the study were previously treated with topical betamethasone + calcipotriol and/or topical calcineurin inhibitors and deemed insufficiently controlled by the attending physician before being offered PUVA.

Reported internal malignancies included breast (n = 5), prostate (n = 4), lung (n = 3), colorectal (n = 4), and single cases of bladder, ovarian, endometrial, and gastric cancer. The majority of patients had their cancer diagnosed 2–3 years before enrollment. All were in complete remission of their cancers at baseline.

Most patients were overweight or obese (80%), and several had comorbid hypertension or dyslipidemia. Eight patients (40%) had severe psoriasis (PASI ≥ 20), while twelve (60%) had moderate disease. Sixteen patients completed all three assessments; four missed the 6-month visit. No patient abandoned treatment. Mean cumulative UVA dose was 120 J/cm^2^ (range 92–150). Patients stratified as severe had higher UVA exposure than moderate cases (*p* = 0.02). No statistically significant differences in UVA exposure were found for PASI75 responders vs. nonresponders (*p* = 0.99), nor for PASI90 responders vs. nonresponders (*p* = 0.099). No obvious differences in adherence were noted according to baseline characteristics, but the sample size limits the interpretability of these data.

### 3.2. Overall PASI Response

PUVA led to a marked reduction in total PASI. Mean PASI decreased from 18.6 ± 3.2 at baseline to 5.7 ± 6.0 at the end of treatment (mean change −12.9; *p* < 0.001), translating to a mean relative reduction of about 69%.

Both severity subgroups improved significantly. Patients with severe psoriasis showed a reduction in total PASI from 21.8 ± 0.9 to 4.2 ± 5.6 (mean change −17.6; *p* < 0.001), while patients with moderate psoriasis improved from 16.4 ± 2.1 to 6.6 ± 6.3 (mean change −9.8; *p* < 0.001). There were significant differences in mean change in total PASI between moderate and severe subgroups (*p* = 0.02), reflecting higher baseline burden in the latter subgroup.

At 6 months, mean total PASI remained significantly lower than baseline (6.9 ± 7.1 vs. 18.6 ± 3.2; *p* < 0.001), in spite of partial loss of response compared with the immediate post-treatment evaluation.

### 3.3. Segmental PASI and Lower-Limb Responsiveness at Completion of PUVA

Segmental PASI analysis showed significant improvements in all anatomical regions at the end of PUVA in both severity subgroups (all *p* < 0.001). However, regional responses clearly differed.

At baseline, the trunk was the most affected region (mean PASI 7.1 ± 1.2). After PUVA, the lower limbs became the most affected area (mean PASI 2.2 ± 1.7). In 16 of 20 patients, the lower-limb PASI was the highest among all segmental scores post-treatment. In the remaining 4 cases (patients 8, 9, 15 and 18), the trunk still represented the highest burden to total disease activity. Interestingly, one of these patients had no excess weight or comorbidities, another patient, although obese, had no other comorbidities, while the other 2 had comorbidities (hypertension and diabetes in one case; hypertension and venous insufficiency in the other). On average, lower-limb lesions represented 46.8 ± 8.5% of the total PASI at that time point (Figure 2). The relative contribution of lower limbs to total disease burden did not differ significantly between moderate and severe cases (*p* = 0.645).

When assessed as relative improvement from baseline, lower limbs showed the weakest response: 59.5 ± 30.0% for lower limbs, 72.2 ± 31.2% for trunk, 72.3 ± 40.0% for upper limbs, and 80.4 ± 23.7% for head/neck. Figure 3 shows that lower limbs had a significantly lower relative improvement than head/neck and trunk (both *p* < 0.001) and upper limbs (*p* = 0.014).

These trends remained after stratifying by basal severity and when analyzing PASI75 responders and PASI90 non-responders. In the small subgroups of PASI75 non-responders (n = 4) and PASI90 responders (n = 4), no statistically significant differences between body areas were observed. Interestingly, only 2 patients (6 and 15) did not present the lower limbs as the anatomical area with the least response after PUVA treatment. So, although no formal analysis can be made, we observe that this phenomenon occurs consistently across the analyzed cases.

### 3.4. Six-Month Outcomes

At 6 months after terminating PUVA, there was a statistically significant increase in total PASI compared with the immediate post-treatment assessment (mean absolute increase 4.3 ± 6.7; *p* = 0.020). This relapse was caused by increases in trunk (*p* < 0.001) and lower-limb segmental PASI (*p* = 0.001). Changes between post-treatment and 6-month assessments for head/neck (*p* = 0.115) or upper limbs (*p* = 0.270) were not statistically significant. These trends were likewise shared by patients with moderate and severe baseline disease.

When segmental PASI at 6 months was assessed as a percentage of baseline, all regions remained significantly improved. Mean improvements were: head/neck 71.2 ± 38.3%, trunk 62.7 ± 36.7%, upper limbs 65.4 ± 51.9%, and lower limbs 52.9 ± 35.4%. Improvement was significantly lower in the lower limbs than in each of the other regions (*p* < 0.01 vs. head/neck and trunk; *p* = 0.032 vs. upper limbs).

At 6 months, the lower limbs once more represented the largest portion of total PASI, similar to the post-PUVA distribution (Figure 2). Lower-limb lesions represented 44.1 ± 9.1% of total PASI, significantly more than head/neck (5.2 ± 2.2%; *p* < 0.001) and upper limbs (13.3 ± 7.9%; *p* < 0.001), and slightly more than trunk (37.4 ± 4.5%; *p* = 0.016).

Four patients had almost complete or complete return to baseline PASI after 6 months. These patients were patients 1, 10, 16 and 17 (in Table 1). Patient 1 was obese, while patients 10, 16 and 17 were overweight. Patient 16 had no other comorbidities, while hypertension was documented in patients 1, 10 and 17; venous insufficiency in patients 1 and 17, and diabetes in patient 17 only. These 4 patients had, respectively, a relative reduction in their total PASI from baseline to completion of treatment of 77.7%, 86.4%, 78.8% and 79.3%. As such, all these patients reached PASI75 response, but none achieved PASI90 response. These responses were not numerically inferior to the overall relative response in this population between the same time points (69%).

### 3.5. Impact on Quality of Life

Mean DLQI decreased from 17.2 ± 2.8 at baseline to 5.6 ± 2.6 at the end of PUVA (*p* < 0.001), pointing towards a significant improvement in health-related quality of life. At 6 months (n = 16), DLQI increased slightly compared with immediately after treatment (7.2 ± 2.6; *p* = 0.002 vs. post-treatment) but remained significantly lower than at baseline (*p* = 0.002).

Correlation analyses showed strong associations between DLQI and both total and segmental PASI at all time points (all *p* < 0.001). However, the relative contribution of each body region to DLQI could not be ascertained due to collinearity and sample size limitations.

## 4. Discussion

In this prospective cohort of patients with moderate-to-severe plaque psoriasis and a recent history of internal solid malignancy, PUVA therapy led to substantial improvements in total PASI and DLQI, with partial persistence of response at 6 months. These findings support the role of PUVA as a valuable non-immunosuppressive option in a population in whom therapeutic options are limited and used cautiously [1,2,3].

The magnitude of PASI reduction observed in this study (approximately 70%) is consistent with historical data from PUVA trials in broader psoriasis populations, which reported PASI reductions of 70–90% in selected patients [6,7]. This is notable given the relatively advanced age, long disease duration, and cancer history of our cohort.

An important contribution of this study is the systematic analysis of the regional response. While all segmental PASI scores improved significantly, lower-limb psoriasis consistently showed the smallest relative reduction, the highest residual burden at the end of PUVA, and the greatest relative contribution to relapse at 6 months. This pattern was consistent across baseline severity groups and responder categories and suggests that the lower limbs represent a site of relative treatment resistance in this context. Furthermore, the same phenomenon has been observed in an analysis of a large dataset of clinical trial patients treated with either secukinumab or ustekinumab. In both arms, lower limbs were shown to be more recalcitrant and slower to respond, compared to other body areas [5]. Recent data on refractory lesions in patients under biologic therapy for plaque psoriasis in Japanese patients further supports our findings [8].

We hypothesize that lower limbs may be a more recalcitrant area to treat in psoriasis due to multifactorial causes, as each individual risk factor alone does not seem to be either necessary or sufficient to account for the reduced therapeutic response. Indeed, several pathophysiological mechanisms may explain the reduced responsiveness of lower-limb psoriasis. The lower limbs are uniquely subject to increased hydrostatic pressure and venous stasis, particularly in the elderly and overweight populations. Chronic venous insufficiency and localized edema are known to impair the clearance of pro-inflammatory mediators and metabolic by-products, and case-based evidence suggests that correction of venous incompetence can lead to marked local improvement or remission of psoriatic plaques [9]. Anatomical variations in skin thickness among body areas may also play a role. The skin of the lower limbs, particularly the pretibial area, possesses a thicker stratum corneum. In psoriatic plaques, this is exacerbated by profound hyperkeratosis and parakeratosis. This increased physical barrier may act as an optical filter, attenuating the penetration of UVA radiation [6].

The lower extremities are areas of high mechanical stress and frequent friction from clothing. These repetitive minor traumas can trigger the Koebner phenomenon, which maintains the hyperproliferative state of the keratinocytes [10]. In patients with a high BMI, increased skin-fold friction and mechanical load may further stimulate the production of “danger-associated molecular patterns” (DAMPs), which can bypass or override the pro-apoptotic effects of PUVA therapy, leading to the recalcitrant clinical phenotype observed in our study.

Finally, recent evidence may indicate that psoriasis is not a molecularly uniform disease across the body. Recent spatial transcriptomic profiling has identified site-specific signatures in psoriatic lesions, including differential expression of genes involved in tissue remodeling and innate immune activation [11]. This distinct molecular profile suggests that lower-limb psoriasis may have a direct and intrinsic predisposition for increased persistence, requiring higher cumulative doses of radiation or adjunctive topical therapy to achieve clearance.

Emerging evidence may further help in understanding this phenomenon.

In our cohort, 80% of patients were overweight or obese. Obesity has been associated with reduced therapeutic response across several psoriasis treatments, including biologics and phototherapy, possibly via altered pharmacokinetics and chronic low-grade inflammation [12,13,14]. Interestingly, the patients who showed marked relapse at 6 months after stopping treatment were all either overweight or obese, and this relapse appears not to be related to relative response at completion of PUVA, as all of them had numerically higher relative improvements than the average of the total study population. Also of interest, one of the patients had no other comorbidity other than excess weight. While the sample size does not allow for subgroup analysis, one may postulate that excess weight might be an important risk factor for disease relapse after PUVA therapy in these patients. The small study sample precludes multivariate analysis, which could be carried in future lines of investigation to ascertain the contribution of each of these phenomena towards the clinical evidence of more recalcitrant disease in the lower limbs.

The strong correlation between DLQI and PASI at all time points underscores the clinical relevance of both overall and regional disease burden. Previous studies have shown that involvement of functionally critical or visible areas—such as legs, hands, feet, scalp, or genitals—can have a disproportionate impact on quality of life relative to total body surface area [15,16]. Our findings support the routine integration of regional assessment into clinical evaluation and suggest that specific attention should be given to lower-limb disease when planning and monitoring treatment. Furthermore, the combined assessment of objective burden of disease and impact on quality of life may be of particular relevance for the choice of the best treatment for each individual patient, better than each variable alone, leading towards a more precise and tailored management of psoriasis.

From a safety point of view, PUVA is known to be associated with an increased risk of non-melanoma skin cancer at high cumulative doses and after prolonged follow-up. In our study, cumulative UVA doses were moderate and within ranges generally considered acceptable for short-term treatment courses [3,7]. No patients experienced acute toxicity and all were instructed to carry the appropriate preventive measures (adequate protective sunglasses after taking the psoralen, photoprotection outside the context of treatment, skin self-assessment, etc.). When multiple courses are anticipated, photoprotective measures are paramount to reduce the risk of skin cancer. In patients deemed to have a high risk of developing non-melanoma skin cancer (previous history of this condition, significant sun damage, previous diagnosis of pre-malignant conditions such as actinic keratosis or actinic cheilitis, etc.) regular follow-up (yearly or every 3 to 6 months according to risk and rate of appearance of premalignant lesions) was offered.

In the context of patients with a recent history of internal malignancy, PUVA offers an important non-immunosuppressive alternative when biologics and conventional systemic agents are restricted or deferred [1,2,3]. Long-term vigilance for skin cancer remains essential, particularly in those who undergo multiple courses of photochemotherapy. When disease is refractory, limited options are available in this subset of patients with a recent history of internal malignancy. Topical treatments may be added but may provide unsatisfactory improvement. We note that in this study, patients were allowed to use topical treatments during the entire protocol according to the physician’s judgment without significant success in overcoming the refractoriness of lesions on the lower limbs. Acitretin may be added to PUVA therapy in a strategy often coined as RePUVA (retinoids + PUVA). By improving hyperkeratosis and theoretically facilitating penetration of the radiation in the skin, this strategy may augment response without raising significant concerns related to internal malignancy. Future studies may address this hypothesis to identify possible strategies to overcome the biological resistance of psoriatic lesions on the lower limbs.

This study has several limitations. The sample size was insufficient to allow for subgroup analyses or a robust multivariable modeling of predictors of response. The absence of a control or comparator group does not allow comparison with other treatment options such as narrowband UVB, conventional systemic treatments, or biologics. Follow-up was limited to 6 months, and cumulative phototherapy exposure over longer periods was not assessed. Staging of the previous internal malignancy and their respective treatment modalities were not accounted for in this study. Future studies may address the potential contribution of these putative factors to the observed effect in larger samples, ideally in multicenter studies, which may offer added granularity and allow generalization to the results presented herein. Nevertheless, the internal consistency of total, segmental, and quality-of-life outcomes and the prospective design strengthen the validity of our observations.

Artificial Intelligence (AI) has shown great promise as an adjunct to clinical assessment in Medicine in general, as well as in skin conditions in particular [17,18]. In psoriasis, such systems could help clinicians flag persistent lower-limb erythema, scale, plaque thickness, and early relapse that are disproportionate to improvement elsewhere, thereby supporting closer monitoring and earlier treatment intensification for anatomically recalcitrant disease. In our opinion, AI could in the future complement segmental PASI scoring by highlighting subtle regional change over time, improving documentation, reducing assessment bias between different observers and helping prioritize follow-up in patients whose lower-limb lesions remain disproportionately active after PUVA.

## 5. Conclusions

PUVA photochemotherapy produced robust improvements in total PASI and quality of life in patients with moderate-to-severe plaque psoriasis and a recent history of internal organ malignancy. These benefits persisted partially at 6 months. However, detailed segmental PASI analysis showed that lower-limb psoriasis is relatively recalcitrant to PUVA and contributes disproportionately to residual disease burden and relapse.

These findings support the use of regional PASI assessment in clinical practice and suggest that adjunctive or targeted strategies may be required to optimize control of lower-limb psoriasis in this complex patient population. Future studies with larger cohorts, longer follow-up, and comparative designs are needed to confirm these results and allow for region-specific treatment approaches.

## Figures and Tables

**Table 1:** Population characteristics of the study cohort (n = 20).

Patient	Age (Years)	Sex	Psoriasis Duration (Years)	Cancer Type	Time Since Cancer Diagnosis (Years)	Cumulative UVA Dose (J/cm^2^)	Body Weight (kg)	BMI (kg/m^2^)	Comorbidities
1	62	M	18	Lung	3	110	92	30.0	HTA, VI
2	55	F	10	Breast	1	125	68	25.9	HTA, DM, VI
3	48	M	7	Prostate	4	95	85	26.8	HC
4	59	M	14	Bladder	3	140	102	31.5	HTA, VI
5	66	F	25	Colorectal	2	118	60	23.4	None
6	71	F	30	Ovarian	3	102	72	26.4	HTA
7	52	M	5	Lung	2	130	78	26.4	HTA, VI
8	64	F	20	Endometrial	2	150	80	28.3	HTA, DM
9	57	M	9	Colorectal	3	112	95	30.3	None
10	69	M	22	Prostate	2	98	88	27.2	HTA
11	50	F	12	Breast	3	121	70	26.3	None
12	73	M	28	Colorectal	2	115	77	25.1	HTA, DM
13	61	F	17	Colorectal	3	145	62	22.2	None
14	46	M	6	Gastric	3	100	74	25.6	VI
15	54	M	11	Prostate	2	110	98	30.9	HTA, VI
16	49	F	8	Breast	2	133	68	25.0	None
17	63	M	15	Lung	2	92	76	25.7	HTA, DM, VI
18	40	F	4	Breast	3	148	58	22.7	None
19	58	M	13	Prostate	2	109	100	30.9	HTA, VI
20	45	F	6	Breast	3	135	72	25.5	VI

Abbreviations: HTA—arterial hypertension; DM—diabetes mellitus; VI—venous insufficiency; HC—hypercholesterolaemia; BMI—body mass index.

## Data Availability

The original contributions presented in this study are included in the article/Supplementary Material. Further inquiries can be directed to the corresponding author.

## Supplementary Materials

The following supporting information can be downloaded at: https://www.mdpi.com/article/10.3390/jcm15124525/s1, Table S1: PASI outcomes per patient and time point. *6m*—6 months after completing PUVA; *A*—At completion of PUVA; *B*—Baseline; HN—Head and Neck; LL—Lower Limbs; PASI—Psoriasis Area Severity Index; Pt—Patient Number; PUVA—Psoralen and Ultraviolet A radiation treatment; T—Trunk; UL—Upper Limbs. DLQI outcomes per patient and time point. Table S2: STROBE Statement.
