# Supplementary material for: Segmental PASI Evaluation Reveals Reduced PUVA Responsiveness of Lower-Limb Psoriasis in Patients with Internal Organ Malignancy
Source: J Clin Med. 2026 Jun 11;15(12):4525. doi: 10.3390/jcm15124525 (PMC13302393; doi:10.3390/jcm15124525)
Supplement: Supplementary file 1 [file jcm-15-04525-s001.zip › Table S1. SuppData.pdf]

Table S1. PASI outcomes per patient and time point. *6m* – 6 months after completing PUVA; *A* – At completion of PUVA; *B* – Baseline; HN – Head and Neck; LL – Lower Limbs; PASI – Psoriasis Area Severity Index; Pt – Patient Number; PUVA – Psoralen and Ultraviolet A radiation treatment; T – Trunk; UL – Upper Limbs.

Pt	Total PASI *B*	HN *B*	T *B*	UL *B*	LL *B*	Total PASI *A*	HN *A*	T *A*	UL *A*	LL *A*	Total PASI *6m*	HN *6m*	T *6m*	UL*6m*	LL *6m*
1	18,4	1,8	7	4,1	5,5	4,1	0,2	1	0,8	2,1	15,7	1,4	5,7	3,9	4,7
2	21,7	2,1	8,4	4,8	6,4	1,3	0,1	0,3	0,3	0,6	1,8	0,1	0,6	0,3	0,8
3	15,3	1,5	5,6	3,4	4,8	3,4	0,2	1	0,4	1,8	4	0,1	1,6	0,2	2,1
4	22	2,2	8,4	4,8	6,6	2,1	0,1	0,8	0,1	1,1	2,7	0,1	1,1	0,2	1,3
5	17,2	1,7	6,5	3,9	5,1	15,1	1,1	4,7	3,4	5,9	*lost*	*lost*	*lost*	*lost*	*lost*
6	14,9	1,5	5,6	3,1	4,7	2,9	0,2	0,8	0,5	1,4	2,8	0,2	1,1	0,2	1,3
7	23	2,3	8,7	5,1	6,9	2,2	0,1	0,8	0,2	1,1	2,9	0,1	1,1	0,4	1,3
8	20,5	2	7,9	4,5	6,1	3	0,1	1,3	0,4	1,2	3,6	0,1	1,7	0,3	1,5
9	17,9	1,7	6,8	4	5,4	19,1	1,5	6,9	5,4	5,3	*lost*	*lost*	*lost*	*lost*	*lost*
10	21,3	2,1	8,3	4,7	6,2	2,9	0,1	1	0,3	1,5	21,6	1,8	8,7	4	7,1
11	22,8	2,3	8,6	5	6,9	2,8	0,1	0,9	0,3	1,5	3,2	0,1	1,2	0,3	1,6
12	13,7	1,2	5,3	3	4,2	2,8	0,2	0,9	0,3	1,4	3,6	0,2	1,4	0,3	1,7
13	16,4	1,6	6,2	3,6	5	2,4	0,1	0,8	0,2	1,3	3,7	0,1	1,5	0,2	1,9
14	12,9	1,1	5	2,9	3,9	2,6	0,2	0,8	0,4	1,2	3,1	0,2	0,9	0,3	1,7
15	19,5	1,9	7,5	4,3	5,8	16,7	1,2	7,5	2,8	5,2	*lost*	*lost*	*lost*	*lost*	*lost*
16	18,9	1,8	7,3	4,1	5,7	4	0,2	1,1	0,5	2,2	20,8	1,5	7,9	5,4	6
17	14,5	1,1	5,6	3,2	4,4	3	0,2	1	0,3	1,5	16,5	1,3	5,3	5,1	4,8
18	22,3	2,3	8,3	4,9	6,8	18,1	0,5	6,1	5,8	5,7	*lost*	*lost*	*lost*	*lost*	*lost*
19	16,8	1,6	6,7	3,5	5	3,5	0,2	1,1	0,5	1,7	2,7	0,1	0,8	0,3	1,5
20	20,7	2	8	4,4	6,3	1,4	0,1	0,4	0,1	0,8	2,1	0,1	0,8	0,2	1

DLQI outcomes per patient and time point

Patients	DLQI Baseline	DLQI After PUVA	DLQI 6m
1	16	3	17
2	19	2	3
3	14	3	4
4	20	2	3
5	15	15	Lost
6	15	3	4
7	21	2	4
8	17	3	5
9	18	16	Lost
10	19	2	17
11	22	3	5
12	13	4	3
13	16	3	4
14	12	3	3
15	18	17	Lost
16	17	4	18
17	15	4	18
18	21	18	Lost
19	16	3	3
20	19	2	4

6m – 6 months after completing PUVA ; DLQI – Dermatological Life Quality Index; PUVA – psoralen and ultraviolet A therapy
