# Supplementary material for: Segmental PASI Evaluation Reveals Reduced PUVA Responsiveness of Lower-Limb Psoriasis in Patients with Internal Organ Malignancy
Source: J Clin Med. 2026 Jun 11;15(12):4525. doi: 10.3390/jcm15124525 (PMC13302393; doi:10.3390/jcm15124525)
Supplement: Supplementary file 1 [file jcm-15-04525-s001.zip › Table S2. STROBE Statement.pdf]

**STROBE Statement—Checklist of items that should be included in reports of cohort studies**		
**Item**	**Recommendation**	**Location**
1a	Indicate study design in title or abstract	Title; Abstract
1b	Informative and balanced abstract	Abstract
2	Background/rationale	Introduction
3	Objectives	Introduction (final paragraph)
4	Study design presented early	Methods – Study Design and Setting
5	Setting, locations, relevant dates	Methods – Study Design and Setting
6a	Eligibility criteria, sources, methods of selection	Methods – Participants
6b	Methods of follow-up	Methods – Participants; Figure 1
7	Clearly defined variables	Methods – Outcomes and Measurements
8	Data sources and measurement methods	Methods – Outcomes and Measurements
9	Efforts to address bias	Methods – Bias and Study Size
10	Study size explanation	Methods – Bias and Study Size
11	Handling of quantitative variables	Methods – Statistical Analysis
12a	Statistical methods, including confounding	Methods – Statistical Analysis
12b	Subgroup analyses	Methods – Statistical Analysis; Results
12c	Handling of missing data	Methods – Statistical Analysis
12d	Loss to follow-up	Methods – Participants; Figure 1
13a	Numbers at each stage	Results – Patient Characteristics; Figure 1
13b	Reasons for non-participation	Results – Patient Characteristics; Figure 1
13c	Flow diagram	Figure 1
14a	Descriptive data	Results – Patient Characteristics; Table 1
14b	Missing data	Results – Patient Characteristics

15	Outcome data	Results – PASI response; Segmental PASI; DLQI
16	Main results with estimates and precision	Results – all subsections
17	Other analyses (subgroups, interactions)	Results – Segmental PASI; Six-month outcomes
18	Key results summarized	Discussion – opening paragraph
19	Limitations	Discussion – final paragraphs
20	Interpretation	Discussion – throughout
21	Generalizability	Discussion – final paragraph
22	Funding	Disclosure (no funding)
23	Ethical approval	Ethics section
